# Prevalence of video gaming disorder in Saudi Arabia: a school-based national study

**DOI:** 10.1186/s42506-024-00165-9

**Published:** 2024-08-21

**Authors:** Amjad Alfaleh, Abrar Alzaher, Abdullah Alkattan, Khaled Alabdulkareem, Mona H. Ibrahim

**Affiliations:** 1 Research and Planning Unit, General Directorate of School Health, Ministry of Health, Riyadh, Saudi Arabia; 2 Department of Research, Assisting Deputyship for Primary Health Care, Ministry of Health, Riyadh, Saudi Arabia; 3 Department of Family Medicine, College of Medicine, Al-Imam Mohammad Bin Saud Islamic University, Riyadh, Saudi Arabia; 4 Department of Public health and Community Medicine, Zagazig University, Zagazig, Egypt

**Keywords:** Gaming disorder, Video gaming, School adolescents, Addiction, Saudi Arabia

## Abstract

**Background:**

Video gaming is a popular leisure activity among adolescents. Those who play excessively are in danger of educational and social drawbacks and may become addicted to video gaming. Several published studies determined the prevalence of GD among children in specific Saudi regions. However, the current study assessed the national prevalence of video gaming disorder (GD) and its risk factors among school students in Saudi Arabia.

**Methods:**

A school-based survey was conducted among adolescents in all regions of Saudi Arabia during the academic year 2021–2022. A multistage stratified cluster sampling technique was used to select the school students. An Arabic-validated version of the 9-item dichotomous (yes/no) GD Scale based on the DSM-5 criteria was used to determine GD prevalence among the students. The score ranged from zero to nine (0–9). Participants who scored five or more were deemed as having GD. Students who scored less than five were classified as normal gamers (score 0–1) or risky gamers (score 2–4).

**Results:**

We recruited 5332 school students. Their mean age was 15.5 ± 1.7 years, and almost half of them were males (50.7%). According to the GD score, the prevalence of normal gamers was 39.08% (*N* = 1714), risky gamers 40.47% (*N* = 1775), and those with GD was, 20.45% (*N* = 897). Logistic regression was performed to determine the association between video gaming disorder and all the gathered variables, which include age, educational grade, sex, types of video gaming, and categories of video games played. The results showed that nationality, age, educational grade, sex, using only mobile devices to play, and playing puzzle and sports games were not associated with video gaming disorder. On the other hand, it was revealed that using tablets, game consoles, PCs; having multiple devices; and playing online, fighting, car racing, war, and adventure games were significantly linked to GD.

**Conclusion:**

The prevalence of GD was 20.45% among Saudi school students who play video games. Utilizing more than one type of gaming device and playing games in the fighting, war, and multiplayer categories via an online connection were significantly linked to having GD. To limit video gaming addiction, we encourage screening, diagnosing, and treating disordered video gamers early. In addition, governmental authorities and video game companies should discuss and revise numerous policy measures to minimize the accessibility of video games, limit the harms and risks related to them, and assist video gamers in becoming effective members of society.

## Introduction

The ubiquity of digital technologies, such as smartphones and tablet computers, has exposed most of the population in modern societies to entertainment software in the form of casual video games or gamified applications [1]. Video games are defined as games played through an electronic system supported by a screen, such as a personal computer (PC), game console, smartphone, or tablet. Playing video games is a popular leisure activity among various age groups, particularly teenagers [2].

Although some reports have claimed that video gaming may be linked to enhanced cognitive functions [3], it was confirmed that video gaming is associated with several negative consequences, including exposure to graphic violence, and poor health [4, 5]. These negative impacts are highly correlated with unrestricted gaming, which could lead to gaming addiction or gaming disorder (GD) for vulnerable individuals [6–8]. Besides, excessive video gamers are at a higher risk of lower educational and professional fulfillment as well as facing more social issues [9–12].

The 11th revision of the International Classification of Diseases (ICD-11), which was released by the World Health Organization (WHO) and the American Psychiatric Association (APA) defines GD as video gaming with the following behaviors: 1) impaired control over gaming (e.g., onset, frequency, intensity, duration, and termination); 2) increasing priority given to gaming to the point where gaming takes precedence over other life interests and daily activities, and 3) continuation of gaming despite the occurrence of negative consequences. These three points are the main diagnostic criteria for GD [13]. GD diagnosis is based on the presence of five or more of the following nine conditions within 12 months: preoccupation, withdrawal, tolerance, loss of control, giving up other activities, continuing despite problems, deception, escaping adverse mood, and relationship loss [14].

There are several published studies that determined the prevalence of GD among children in specific Saudi regions [15–18]. However, the current study assessed the national prevalence of GD and its risk factors among adolescent school students in Saudi Arabia.

## Methods

### Study design and population

A school-based cross-sectional study was conducted during the academic year 2021–2022 among adolescent school students (age range 12 to 18 years old) in all Saudi regions. The study included school students of both sexes (male and female) attending middle and high schools. Students whose guardians refused to enroll them in the study and students attending international schools (e.g., schools for specific nationalities) were excluded.

### Sample size and technique

The sample size was calculated, taking into account the number of students (2,519,150) at middle and high schools in Saudi Arabia in 2020 [19], the variability of video gaming disorder prevalence revealed by previous studies in different Saudi regions, and the prevalence (80.7%) of video gaming revealed by Awadalla et al. [15]. Therefore, we considered a precision of 3%, a confidence level of 97%, and a design effect of 3 to assure a high degree of certainty in the outcomes, the generalizability of the included population, and overcoming cluster sampling error. Based on the sample size calculation, the minimum sample size was 881 students.

A multistage stratified cluster sampling technique was used to select the participants. A simple random method was used to select 199 schools from a list that includes all middle and high schools in Saudi regions. This method allowed for the inclusion of 168 governmental and 31 private schools, of which 104 were middle and 95 were high schools. A stratified cluster random sampling was followed to select one class from each grade within each school randomly. The proportional allocation of male and female students and the number of governmental and private schools was considered.

### Study tools and data collection

The questionnaire consists of three sections. The first section involves four open-ended demographic questions that are utilized to gather students’ demographic data, including age, sex, nationality, and educational grade. Before the students start to complete the next sections, a yes/no question about their video gaming status was utilized to determine whether they were non-video gamers or video gamers. The questionnaire indicates that students who describe themselves as non-video gamers should not complete the next sections. The second section consists of two multiple-choice questions (in the form allowing the selection of more than one response to the given options) that focus on the types of gaming and game categories used by the students. The third section consists of an Arabic-validated version of the 9-item dichotomous (yes/no) GD scale based on the DSM-5 criteria used to determine GD prevalence among the students. The score ranges from zero to nine (0–9). Participants who scored five or more were recognized as having GD. Students who scored less than five were either classified as normal gamers (score 0–1) or risky gamers (score 2–4) [20–22].

A pilot study was conducted to test the data collection logistics and the clarity of the data collection tool. As a result, two questions were modified. Pilot study data was excluded from the final study.

### Statistical analysis

The data were collected from 104 middle schools and 95 high schools via trained coordinators in each Saudi region. Trained coordinators in each Saudi region were responsible for data collection and data entry into the unified electronic website established by the Saudi Ministry of Health. The collected and entered data were cleaned, coded, and analyzed using Microsoft Excel and SPSS software version 25 [SPSS Inc., Chicago, IL, USA]. Descriptive statistics were presented as frequencies and percentages for categorical data and mean and standard deviation (SD) for continuous data. The chi-square (χ2) test and ANOVA test were used to determine the association between categorical and continuous variables, respectively. Moreover, factors that were significantly associated with students having GD were further analyzed using logistic regression, hence, the values of pseudo R-squared (Nagelkerke R-squared), estimated coefficient (B), and odds ratios with a 95% confidence interval (CI) were determined. The odds ratios were described as exponential values of B (Exp(B)).

### Ethical considerations

The study was approved by the Institutional Review Board of the Saudi Ministry of Health. Permission from school authorities and written consent or assent were obtained.

## Results

We recruited 5332 middle (52.6%) and high (47.4%) school students. Their mean age was 15.5 ± 1.7 years; most of them were Saudi citizens (89.9%), and nearly half of them were males (50.7%). The overall percentage of students playing video games was 82.2% (*N* = 4385), which significantly consisted of students who were younger in age, males, Saudi citizens, and at lower educational grades (*p* < 0.001). Students identified as non-video gamers (*N* = 947, 17.8%) were not eligible to fill out the GD scale (Table 1).

**Table 1:** Characteristics of the studied school students based on video gaming status (non-video gamers versus video gamers), Saudi Arabia, 2021–2022

Variable	Total Participants (*N* = 5332)	Students video gaming status		*P*-value
		**Non-video gamers (*N* = 947)**	**Video gamers** **(*N* = 4385)**	
**Age in years (Mean ± SD)**	15.45 ± 1.72	15.71 ± 1.74	15.39 ± 1.71	< 0.001 *
**Boys N (%)**	2701 (50.7)	340 (12.6)	2361 (87.4)	< 0.001^δ^
**Girls N (%)**	2631 (49.3)	607 (23.1)	2024 (76.9)	
**Saudi citizens N (%)**	4797 (89.9)	811 (16.9)	3985 (83.1)	< 0.001^δ^
**Saudi residents** N (%)	536 (10.1)	136 (25.4)	400 (74.6)	
**Middle school students N (%)**	2804 (52.6)	441 (15.7)	2363 (84.3)	< 0.001^δ^
**High school students N (%)**	2528 (47.4)	506 (20.0)	2022 (80.0)	

^*^Student’s T-test

^δ^Chi-square test

According to the GD score, the prevalence of normal gamers was 39.08% (*N* = 1714), risky gamers 40.47% (*N* = 1775), and those with GD was, 20.45% (*N* = 897). There were no significant differences among the three groups of gamers regarding age (15.37 ± 1.72, 15.38 ± 1.72, and 15.46 ± 1.68 years, respectively, *p* = 0.198), Saudi citizens vs. residents (38.9% vs. 41.0%, 40.7% vs. 38.0%, and 20.4% vs. 21.0%, respectively, *p* = 0.562), and middle vs. high educational grades (38.7% vs. 39.5%, 41.7% vs. 39.1%, and 19.6% vs. 21.4%, respectively, *p* = 0.169). Although disordered gaming was not associated with sex, risky gaming was significantly associated with sex compared to normal gaming, the proportion of male risky gamers was higher than that of females (*p* < 0.001) (Table 2).

**Table 2:** Students’ characteristics, type of gaming, and games categories played among normal, risky, and disordered gamers, Saudi Arabia, 2021–2022

^Students’ characteristics^	Total video gamers (*N* = 4385)	Normal gamers (Score = 0–1) (*N* = 1713)	Risky gamers (Score = 2–4) (*N* = 1775)	Disordered gamers (Score = 5 +) (*N* = 897)	*P-value
**Age (mean ± SD)**	15.39 ± 1.71	15.37 ± 1.72	15.38 ± 1.72	15.46 ± 1.68	0.198 *
**Boys N (%)**	2361 (53.8)	863 (36.5)	1015 (43.0)	483 (20.5)	< 0.001 ^δ α^
**Girls N (%)**	2024 (46.2)	850 (42.0)	760 (37.5)	414 (20.5)	
**Saudi citizens N (%)**	3985 (90.9)	1549 (38.9)	1623 (40.7)	813 (20.4)	0.562 ^δ^
**Saudi residents N (%)**	400 (9.1)	164 (41.0)	152 (38.0)	84 (21.0)	
**Middle school students N (%)**	2363 (53.9)	915 (38.7)	984 (41.7)	464 (19.6)	0.169 ^δ^
**High school students N (%)**	2022 (46.1)	798 (39.5)	791 (39.1)	433 (21.4)	
^**Ω**^**Play via PC N (%)**	693 (15.8)	190 (27.4)	311 (44.9)	192 (27.7)	< 0.001 ^δ β^
^**Ω**^**Play via game console N (%)**	2520 (57.5)	863 (34.2)	1090 (43.3)	567 (22.5)	< 0.001 ^δ ¶^
^**Ω**^**Play via tablet N (%)**	502 (11.4)	153 (30.5)	183 (36.4)	166 (33.1)	< 0.001 ^δ ‡^
^**Ω**^**Play via smartphone N (%)**	2901 (66.2)	1160 (40.0)	1130 (39.0)	611 (21.0)	0.015 ^δ γ^
^**Ω**^**Play via multiple devices N (%)**	1663 (37.9)	529 (31.8)	705 (42.4)	429 (25.8)	< 0.001 ^δ κ^
**Online video gaming N (%)**	4055 (92.5)	1525 (37.6)	1674 (41.3)	856 (21.1)	< 0.001 ^δ ε^
^**Ω**^**Fighting games N (%)**	2094 (47.8)	615 (29.4)	943 (45.0)	536 (25.6)	< 0.001 ^δ τ^
^**Ω**^**Car racing games N (%)**	1179 (26.9)	406 (34.4)	500 (42.4)	273 (23.2)	< 0.001 ^δ ω^
^**Ω**^**Sports games N (%)**	1457 (33.2)	572 (39.3)	569 (39.1)	316 (21.7)	0.090 ^δ λ^
^**Ω**^**War games N (%)**	1140 (26.0)	285 (25.0)	519 (45.5)	336 (29.5)	< 0.001 ^δ θ^
^**Ω**^**Adventure games N (%)**	1842 (42.0)	631 (34.3)	780 (42.3)	431 (23.4)	< 0.001 ^δ π^
^**Ω**^**Puzzle games N (%)**	1172 (26.7)	477 (40.7)	443 (37.8)	252 (21.5)	0.080 ^δ ψ^
^**Ω**^**Multiplayer games N (%)**	1690 (38.5)	520 (30.8)	739 (43.7)	431 (25.5)	< 0.001 ^δ μ^

^Ω^Overlapped answers (more than one answer per student)

^*^ANOVA test performed

^δ^Chi-square test performed

^α^Boys vs. girls: Risky gaming was significantly associated with sex compared to normal gaming (*p* < 0.001)

^β^Play via PC: Disordered gaming was significantly associated with PC use compared to normal and risky gaming (*p* < 0.001)

^¶^Play via game console: Disordered and risky gaming were significantly associated with game console use compared to normal gaming (*p* < 0.001)

^‡^Play via tablet: Disordered gaming was significantly associated with tablet use compared to normal and risky gaming (*p* < 0.001)

^γ^Play via smart mobile: Disordered gaming was significantly associated with smart mobile use compared to risky gaming (*p* = 0.015)

^κ^Play via multiple devices: Disordered gaming was significantly associated with multiple devices use compared to normal and risky gaming (*p* < 0.001)

^ε^Online video gaming: Disordered gaming was significantly associated with online video gaming compared to normal gaming (*p* < 0.001)

^τ^Fighting games: Disordered gaming was significantly associated with fighting games compared to normal and risky gaming (*p* < 0.001)

^ω^Car racing games: Disordered gaming was significantly associated with car racing games compared to normal gaming (*p* < 0.001). λ Sports games: Disordered gaming was not significantly associated with sports games compared to normal and risky gaming (*p* = 0.090)

^θ^War games: Disordered gaming was significantly associated with war games compared to normal and risky gaming (*p* < 0.001)

^π^Adventure games: Disordered gaming was significantly associated with adventure games compared to normal and risky gaming (*p* < 0.001)

^ψ^Puzzle games: Disordered gaming was not significantly associated with puzzle games compared to normal and risky gaming (*p* = 0.080)

^μ^Multiplayer games: Disordered gaming was significantly associated with multiplayer games compared to normal and risky gaming (*p* < 0.001)

Table 2 also presents the frequencies and percentages concerning the type of gaming and categories of video games played by normal, risky, and disordered gamers. Based on the chi-squared test, disordered gaming was not significantly associated with school students who played sports (*p* = 0.090), and puzzle (*p* = 0.080) video games. However, significant associations were found between disordered and non-disordered gamers (either normal or risky gamers) regarding utilizing PCs, tablets, game consoles, mobile devices, and multiple devices for video gaming (*p* < 0.001). In addition, playing online, fighting, car racing, war, adventure, and multiplayer video games were also statistically linked to video gaming disorder (*p* < 0.001).

Additionally, logistic regression was performed to ascertain the association between video gaming disorder and all the gathered variables, which include age, educational grade, sex, types of video gaming, and categories of video games played. The results showed that nationality, age, educational grade, sex, using only mobile devices to play, and playing sports and puzzle games were not associated with video gaming disorder (*p* > 0.05) (Table 3). In addition, it was revealed that using tablets, game consoles, PCs, multiple devices, playing online, fighting, car racing, war, and adventure games were significantly linked to GD (Table 3).

**Table 3:** Logistic regression of the relationship between gamers with gaming disorder score > 4 and specific gamers’ characteristics

Student characteristic	Dependent variable: gaming disorder score > 4 (i.e., students with gaming disorder)				
	**Nagelkerke r**^**2**^	**B**	**Exp(B)**	**95% CI for Exp(B)**	***p*-value**
**Nationality**	0.000	0.036	1.037	0.806–1.335	0.777
**Age**	0.001	0.121	1.128	0.974–1.307	0.108
**Educational grade**	0.001	0.109	1.115	0.963–1.292	0.146
**Sex**	0.000	0.000	1.000	0.863–1.158	0.998
**Play via mobile**	0.001	0.111	1.118	0.955–1.307	0.165
**Play via tablet**	0.022	0.756	2.130	1.740–2.609	** < 0.001**
**Play via game console**	0.005	0.300	1.350	1.161–1.571	** < 0.001**
**Play via PC**	0.010	0.485	1.624	1.349–1.955	** < 0.001**
**Play via multiple devices**	0.014	0.515	1.674	1.444–1.942	** < 0.001**
**Play online video games**	0.006	0.635	1.886	1.348–2.638	** < 0.001**
**Play fighting games**	0.020	0.609	1.839	1.584–2.135	** < 0.001**
**Play car racing games**	0.002	0.221	1.247	1.061 -1.465	**0.007**
**Play war games**	0.024	0.693	1.999	1.710–2.338	** < 0.001**
**Play adventure games**	0.005	0.309	1.361	1.175–1.578	** < 0.001**
**Play puzzle games**	0.000	0.087	1.091	0.926–1.285	0.300
**Play sports games**	0.001	0.112	1.119	0.959–1.305	0.154

*GD* Gaming disorder, *B* Estimated coefficient, *Exp(B)* Exponential value of B, which represents odds ratio, *r*^*2*^ R-squared

**Fig. 1:**
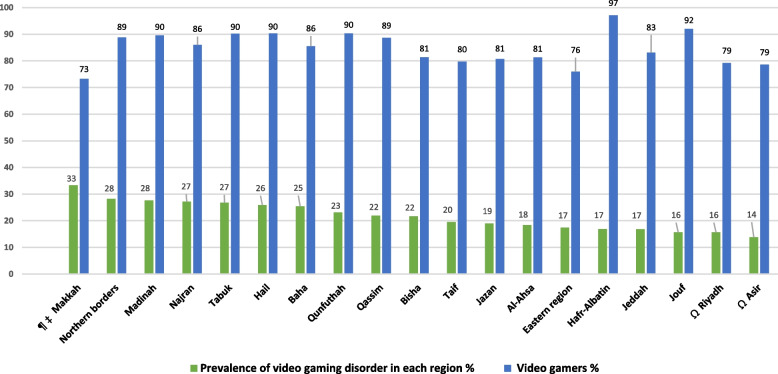
Percentages of video gamers and prevalence of video gaming disorder in each Saudi region, 2021–2022 Chi-square test performed to compare GD prevalence between Saudi regions. ¶ Differences in video gaming disorder prevalence between Makkah, the Northern Borders, Madinah, Najran, Tabuk, Hail, Baha, and Qunfuthah regions were not significant (*p* > 0.05). ‡ The prevalence of video gaming disorder in Makkah region was significantly higher than that of Qassim, Bisha, Taif, Jazan, Al-Ahsa, Eastern region, Hafr-Albatin, Jeddah, Jouf, Riyadh, and Asir regions (*p* < 0.05). Ω The prevalence of video gaming disorder in Riyadh and Asir regions was significantly lower than that of the Northern Borders, Madinah,Najran, Tabuk, Hail, Baha, Qunfuthah, Qassim, and Bisha regions

At the regional level, the prevalence of GD ranged from 13.8% to 33.3%. Two regions were recognized as having significantly the lowest percentages of students with GD, including Asir (13.8%) and Riyadh (15.7%) when compared with Makkah (33.3%), Northern borders (28.2%), Madinah (27.6%), Najran (27.2%), Tabuk (26.8%), Hail (25.9%), Baha (25.4%), Qunfuthah (23.1%), Qassim (21.9%), and Bisha (21.7%) regions (Fig. 1).

## Discussion

The current study utilized the 9-item dichotomous GD scale based on the DSM-5 criteria to determine GD prevalence among middle and high school students during the academic year 2021–2022 in all Saudi regions. The total prevalence of GD across the study population was 16.8%, while the prevalence of GD across participants who play video games was 20.45%. Moreover, it was found that students’ characteristics, including age, nationality, sex, and educational grade were not associated with GD. In addition, playing via mobile devices only and playing puzzle and sports games were not linked to GD. However, students who used other playing devices, and other game categories were significantly more likely to have GD.

At the regional level in Saudi Arabia, there were notable variations in the prevalence of gaming disorder (GD) among school students, regardless of whether the percentages of gamers in each region were high or low.

Comparing the current Saudi national GD prevalence with other Saudi studies conducted in specific regions, Alhamoud et al. (2022) [16] found that the prevalence of GD among adolescents was 21.9% in Dammam (a city in the Eastern region), Alfaifi et al. (2022) [17] reported a rate of 29.3% in Faifa (a city in the Jazan region), and Rajab et al. (2020) [18] found a prevalence of 5.1% in Qassim (the Qassim region). There are clear variations between the outcomes found in this study and these three regional studies due to differences in sample size and the GD scoring criteria.

A systematic review and meta-analysis conducted during the 2010s recorded a global GD prevalence among adolescents ranging from 1.04% to 21.76%. [23]. However, subsequent global studies on GD among adolescents showed varying results. For instance, an Indian study conducted among adolescents reported a GD prevalence of 2.6% [24], while Chinese studies reported a GD prevalence of 13%, with a relatively higher rate among males [25, 26]. Among European communities, GD prevalence among German adolescents was 3.5% [27], while it was lower among Russian [28], Spanish [29], Norwegian [30], and Finnish [31] students living in the European part. Similar disparities were reported in Latin American countries, where GD prevalence was 5% and 28% in Mexican and Brazilian adolescents respectively [32, 33]. In the Middle East, a lower prevalence of GD was reported among Egyptian and Iranian students (10.5%) [34, 35]. The prevalence of GD in our study is considered high compared to European countries. However, it is similar to the outcome of studies conducted in Southern East Asia, and some Middle Eastern countries. Variations in the results between our study and the previously mentioned studies could be attributed to technological advances and COVID-19 pandemic environmental factors, i.e., lockdown and distance learning, and cultural differences in gaming among communities. Moreover, the differences in the results could be due to the different study samples, assessment tools, or cutoff points.

Our results regarding game categories played by students with GD are in line with Granic et al. study, which showed that stimulatory, sports, online, and multiple-mode games are commonly played by adolescents with GD and may predict violence among them [3]. Yet, the current study observed that fighting and adventure games were more frequently played than the above-mentioned game categories.

In summary, the prevalence of video game use is very high globally. However, the prevalence of GD hugely varies among different countries. Countries with a high prevalence of GD are encouraged to screen, diagnose, and treat disordered video gamers early. Schools are the most suitable places to screen for GD, after which students can be referred to specialists to confirm the diagnosis [36–38]. In order to limit video gaming addiction, governmental authorities and video game companies have discussed numerous policy measures. These policies aim to minimize the obtainability of video games, limit the harms and risks related to them, and assist video gamers in becoming effective members of society. For instance, the South Korean government restricted non-adult access to the internet during specific night hours. In China, minors are limited to playing video games for one hour a day on specific days. Additionally, the gaming industry uses warning notes and addictiveness ratings on games, which enable parents to be aware of possible risks and choose the least addictive games for their children. Specific centers were established to prevent gaming addiction, such as The Internet Addiction Prevention and Counseling Center in South Korea, the Center for Internet Addiction in the United States, and the Internet Addiction Treatment Center in the General Hospital of Beijing Military Region in China [39]. Moreover, there must be useful alternative activities to occupy adolescents' free time.

### Limitations

The cross-sectional design may not allow for establishing causality between video gaming disorders and risky behavior. Also, the self-reported data gathered from the questionnaire may underestimate or overestimate the phenomenon in this study. Besides, the relationship between video gaming disorder and scholastic achievement was not studied.

## Conclusions

The prevalence of video gaming disorder was 20.45% among Saudi school students who play video games. Utilizing more than one type of gaming device and playing fighting, war, and multiplayer games categories via online connection were significantly linked to students with GD.

To limit video gaming addiction, we encourage screening, diagnosing, and treating disordered video gamers early. In addition, governmental authorities and video game companies should discuss and revise numerous policy measures to minimize the obtainability of video games, limit the harms and risks related to them, and assist video gamers in becoming effective members of society.

## Data Availability

The study's supporting data can be obtained from the corresponding author upon reasonable request (Alkattan A).
